# Editorial: “One Health” Approach for Revealing Reservoirs and Transmission of Antimicrobial Resistance

**DOI:** 10.3389/fmicb.2021.830211

**Published:** 2022-01-17

**Authors:** Ziad Daoud, Jean-Marc Rolain

**Affiliations:** ^1^College of Medicine, Central Michigan University, Mount Pleasant, MI, United States; ^2^Michigan Health Clinics, Saginaw, MI, United States; ^3^Unité de Recherche Microbes Evolution Phylogeny and Infection (MEPHI), Aix-Marseille Université, Assistance Publique- Hopitaux de Marseille, Institut de Recherche pour le Développement, Instituts Hospitalo-Universitaires Méditerranée Infection, Marseille, France

**Keywords:** one health, resistance, CRE, ESBL, environment, animal, human

The goals of medicine have mostly aimed at relieving pain and suffering, promoting health and preventing diseases, as well as curing diseases when possible and providing care when cure is not achievable (Cassell, 1998). Medicine has always been focused on human beings, and this has not changed. However, what has changed, or evolved, is our understanding of the interactions existing between humans and their environments, including nature, animals, societies, and even inanimate objects. The field of Microbiology and Immunology has been pioneering in investigating and understanding how humans react and interact with their environments. Since Edward Jenner (1749–1823) discovered “the cowpox protection against smallpox” (Riedel, 2005) and Louis Pasteur (1822–1895) confirmed the “Germ theory of disease” (Williamson, 1955), scientists have made huge contributions to the field.

Over the last 40 years, Multi-Drug Resistant Gram-Negative Bacilli including Extended-Spectrum Beta-Lactamase (ESBLs), carbapenemase producers, Methicillin-Resistant *Staphylococcus aureus* (MRSA), Vancomycin Resistant Enterococcus (VREs), as well as many other resistant organisms have been extensively reported in patients and humans (Fournier et al., 2012; Stokle et al., 2013; Creighton and Howard, 2017). In this context, data from patients is tremendous and has reached an advanced level of understanding of the molecular mechanisms behind resistance in these bacteria. Resistance has been shown to be disseminated in humans, but also animals, and the environment (Daoud et al., 2018). In many cases, microorganisms isolated from humans and animals share the same mechanisms of resistance (Baquero et al.). Many studies investigating resistance in the environment have also reported resistant bacteria in wastewaters and natural environments. More recently, resistance to colistin has been detected and reported in several hospital and community environments such as animal farms. In 2010, The Food and Agriculture Organization, World Organization for Animal Health, and World Health Organization initiated programs of collaboration for “Sharing responsibilities and coordinating global activities to address health risks at the animal-human-ecosystems interfaces^1^.”

The present Research Topic “”*One Health” Approach for Revealing Reservoirs and Transmission of Antimicrobial Resistance*” shows that understanding the epidemiology, characteristics, mechanisms, and elements of resistance in different microenvironments is imperative for the successful control of the global spread of resistant bacteria and resistance genes between the three components of One Health: Humans, Environment, and Animals.

At the Environment level, the study “*Bacteria From the Multi-Contaminated Tinto River Estuary (SW, Spain) Show High Multi-Resistance to Antibiotics and Point to Paenibacillus spp. as Antibiotic-Resistance-Dissemination Players*” of Eduardo-Correia et al. found that *Paenibacillus* isolates share many resistance mechanisms with several other genera and suggests a role for this bacterium in the inter-genus dissemination of antibiotic resistance. For the first time, a possible hotspot of resistance interchange in a particular environment was detected. This leads to the possibility that one bacterial member of the community could be responsible for the promotion of antibiotic resistance in this environment. Chen et al. show co-occurrence of heavy metals and genes of resistance in a specific environment. The study *Bacterial Heavy-Metal and Antibiotic Resistance Genes in a Copper Tailing Dam Area in Northern China* suggests that heavy Metal Resistance genes (MRGs) and antimicrobial resistance genes (ARGs) can be co-selected in soil contaminated by heavy metals. These findings shed light on the relationship between heavy metals and antimicrobial resistance, a Research Topic where final and clear conclusions have not been yet fully described.

The spread of resistance genes in the environment, specifically in forest and grassland soil, as well as in rivers is addressed in this Research Topic in two studies. The first on the “*Discovery of Novel Antibiotic Resistance Determinants in Forest and Grassland Soil Metagenomes*” of Willms et al. reports for the first time on the presence of non-mobile dihydropteroate synthase (DHPS) genes conferring resistance to sulfonamides in forest soil with no history of exposure to these synthetic drugs. The second “*Emergence and Characterization of a Novel IncP-6 Plasmid Harboring blaKPC*−*2 and qnrS2 Genes in Aeromonas taiwanensis Isolates*” of Hu et al. detected blaKPC−2 and qnrS2 genes on a non-conjugative plasmid in three carbapenem resistant isolates of *Aeromonas taiwanensis* from river sediments samples. These studies conclude that the spread of genes of resistance and resistant organisms in natural environments form a close monitoring system to control the occurrence and spread of such MDR mechanisms.

In parallel, the results of another study assessing the occurrence of ARGs in Waste Water Treatment Plants (WWTPs) “*Whole Genome Sequencing of Extended-Spectrum Beta-Lactamase (ESBL)-Producing Escherichia coli Isolated From a Wastewater Treatment Plant in China*” of Jiang et al. are in full agreement with the previous two studies. The Whole Genome Sequencing results suggest that pharmaceutical WWTP can play a significant role in the emergence of ARGs and suggest the need to strengthen active surveillance of ARB and ARGs from the pharmaceutical industry.

Studies exploring resistance in hospitals and humans are countless, fewer are those exploring animals and nature. More importantly, in view of the geographic and cultural changes imposed by war migrations, an in-depth assessment of the interlinkages existing between humans, animals, and environments is needed. The large use of animal products in human agriculture, the carriage of resistant bacteria, and the spread of antimicrobial resistance in the environment is posing a potential risk for transmitting resistance from poultry and poultry products to the human population. Descriptive studies of prevalence and surveillance of resistant bacteria, identification of new/undescribed reservoirs and mechanisms of resistance, channels of transmission, and subsequent alterations of the flora, are also instrumental in our battle against bacterial resistance.

In addition to the environment, animals constitute the second determinant of One Health. In this context, the use of antibiotics in animal farms is a major issue that needs more attention. A comparative molecular characterization of Enterobacteriaceae carrying Extended-spectrum β-lactamase (ESBL) and AmpC, isolated from human stool samples, rectal swabs from companion animals, and swabs from the environment of veterinarian hospitals in South Korea was performed by Hong et al. The results showed that while in animals blaCMY-2-like, blaCTX-M-55, and blaCTX-M-14 (16.1%) were the most commonly found genes of resistance, blaCTX-M-15 was predominant in human samples. The pulsotypes of *E. coli isolates* from dogs and humans showed more than 85% similarity, suggesting a direct transmission and dissemination of extended spectrum cephalosporin-resistant Enterobacteriaceae between humans and companion animals. Similarly, a study performed on patients hospitalized in Kuwait “*Livestock-Associated Methicillin-Resistant Staphylococcus aureus in Patients Admitted to Kuwait Hospitals in 2016–2017*” of Boswihi et al. identified four Livestock Associated MRSA clones with CC97-MRSA-V [fusC+] as the dominant one, and suggested an independent acquisition from different sources. Another research work from Beijing-China reports the detection of Multi-Drug Resistant *Campylobacter jejuni* from the wild birds “silver pheasant,” which might lead to potential public health threats as vectors of this organism.

Many studies in this Research Topic addressed resistance in animal farms. According to AbuOun et al., animals carrying resistance at the start of the fattening period can be a reservoir and the starting point for the transmission of bacterial resistance to the other animals in the same flock. On the other hand, the study “*Characterizing Antimicrobial Resistant Escherichia coli and Associated Risk Factors in a Cross-Sectional Study of Pig Farms in Great Britain*” reports that *E. coli* isolates from pig farms were “phylogenetically diverse harboring a variety of AMR profiles with widespread resistance to old antibiotics with no concurrent resistance to third-generation cephalosporins, fluoroquinolones, and aminoglycosides.”

Interestingly, Schnaiderman-Torban et al. found a significant correlation between petting zoos and the spread of antibiotics-resistant ESBL/AmpC-producing bacteria, including highly virulent, disease-associated MDR *E. coli* strains. This highlights the importance of having infection control guidelines for such environments where individuals, children, and families interact directly and indirectly with the animals.

Gozi et al. conclude that feedlot lambs act as reservoirs of commensal multidrug-resistant *E. coli*, and antibiotic resistance can reach humans through the food chain. Their study “*Dissemination of Multidrug-Resistant Commensal Escherichia coli in Feedlot Lambs in Southeastern Brazil*” constitutes the first report of a so broad characterization of antimicrobial resistant *E. coli* isolated from sheep.

The Research Topic of antimicrobial use in preventive/curative treatment in farms can have several side effects and can lead to collateral damage. The study by Zeineldin et al. evaluated the impacts of early-life antimicrobial intervention on fecal microbiota development, and the prevalence of selected ARGs in neonatal piglets. Their results demonstrate that the shifts in fecal microbiota structure caused by perinatal antimicrobial intervention are modest and limited to groups of microbial taxa. Maciuca et al. come up with concurring results showing that high prevalence of mcr-1 plasmid mediated colistin resistance in commensal AmpC producing *Escherichia coli* from poultry suggests the selection of these isolates by prophylactic and/or therapeutic farm use of colistin and/or cephalosporins. In this same context, our correspondence to The Lancet Infectious Diseases (Olaitan et al., 2021) concluded that colistin use in farms is leading to a spread of colistin resistance, and suggested that a ban of colistin use as a feed additive for growth promotion would slow or “possibly stop” the spread of plasmid-mediated colistin resistance.

Studies exploring the discovery of new antibacterial molecules are highly needed for treating human and animal infections. In addition, studies involving new means of breaking the cycle of transmission of resistance, are highly encouraged.

## Author Contributions

All authors listed have made a substantial, direct, and intellectual contribution to the work and approved it for publication.

## Conflict of Interest

The authors declare that the research was conducted in the absence of any commercial or financial relationships that could be construed as a potential conflict of interest.

## Publisher's Note

All claims expressed in this article are solely those of the authors and do not necessarily represent those of their affiliated organizations, or those of the publisher, the editors and the reviewers. Any product that may be evaluated in this article, or claim that may be made by its manufacturer, is not guaranteed or endorsed by the publisher.

## Footnotes

^1^ https://www.who.int/influenza/resources/documents/tripartite_concept_note_hanoi/en/
